# *Schistosoma haematobium* Infection and Buruli Ulcer

**DOI:** 10.3201/eid1003.020514

**Published:** 2004-03

**Authors:** Janet T. Scott, Roch C. Johnson, Julia Aguiar, Martine Debacker, Luc Kestens, Augustin Guedenon, Bruno Gryseels, François Portaels

**Affiliations:** * Institute of Tropical Medicine, Antwerp, Belgium; † Centre de Dépistage et de Traitement des Ulcères de Buruli de Lalo, Bénin; ‡ Centre Sanitaire et Nutritionnel Gbemonten, Zagnanado, Bénin; § Ministère de la Santé Publique, Cotonou, Bénin

**Keywords:** *Schistosoma haematobium*, *Mycobacterium ulcerans*, human, Benin

**To the Editor:** Buruli ulcer caused by *Mycobacterium ulcerans* was recognized in 1997 as an emerging public health problem by the World Health Organization (WHO) (*1*). The disease is found in tropical Africa, the Americas, Australia, and Asia (*2*). In Benin, severe disease with serious complications is reported with increasing frequency. Buruli ulcer causes serious deformities and disability, particularly since amputating limbs is sometimes required in cases of severe disease such as osteomyelitis (*3*). Given the effect on the quality of life of those afflicted, and the lack of adequate treatment, identifying host risk factors for Buruli ulcer is an important research imperative (*2*). We investigated one potential risk factor, concurrent infection with *Schistosoma haematobium.* Preliminary data indicate that although *S. haematobium* is not a risk factor for Buruli ulcer, it may be associated with osteomyelitis.

Although Buruli ulcer and schistosomiasis each exist in the absence of the other, close parallels exist between their epidemiology, suggesting that schistosomiasis could be one possible risk factor for Buruli ulcer. Both diseases are associated with the tropical wetlands of west and central Africa. Cases of both schistosomiasis and Buruli ulcer have increased rapidly in these areas since the 1980s, particularly after irrigation and dam construction. Buruli ulcer is most frequent in children <15 years of age; this group typically also has the highest prevalence and intensity of schistosome infections. Schistosomiasis is transmitted through contact with infected water, when the cercarial larvae penetrate skin, and increasing evidence exists that *M. ulcerans* proliferates in the bottom mud of stagnant waters and may be harbored by aquatic insects (*4*).

An immunologic rationale for linking the two diseases has been proposed (*5*). Briefly, protective immune responses to other mycobacterial diseases are known to depend on a type 1 cellular response, typified by interferon-gamma (IFN-γ). Helminth infections, on the other hand, are classically associated with type 2 responses, typified by interleukin (IL)-4 and IL-5 production. Therefore, a concurrent infection with a bloodborne helminth such as *S. haematobium* may skew the immune response away from a potentially protective type 1 response (*5*).

A total of 113 patients were recruited from Buruli ulcer treatment centers in Lalo (Couffo Department) and Zagnanado (Zou Department) in Benin. A team of experienced surgeons clinically confirmed all cases of Buruli ulcer. Controls (n = 429) were recruited at random from residents of eight current Buruli ulcer foci in the Couffo Department. Past or current Buruli ulcer patients were excluded from the lottery for controls.

Clinical records reported no case of schistosomiasis in this area. This finding was confirmed by a preliminary survey of 60 Buruli ulcer patients, which detected no concurrent *S. mansoni* by using the Kato Katz method (*6*). Diagnosis of *S. haematobium* was performed by filtering three urine samples given on different days. Neither cases nor controls were asked to exercise (as is usual) before giving urine samples because many Buruli ulcer cases were immobile. All patients positive for *S. haematobium* were offered praziquantel treatment.

In the entire participating population, 11.5% (95% confidence interval [CI] 6% to 19%) of Buruli ulcer cases were positive for *S. haematobium*; 11.1% (95% CI 5% to 20%) of cases from Lalo and 12.2% (95% CI 4% to 26%) from Zagnanado were positive. The difference between the two centers was not statistically significant. Of the 429 non-Buruli ulcer controls, 9.5% (95% CI 7% to 13%) were positive for *S. haematobium*. No statistically significant difference between cases and controls was detected. The odds ratio for *S. haematobium* infection in a logistic regression model (which also included age and sex) was 1.3 (95% CI 0.63 to 2.4). Prevalence of *S. haematobium* infection did not significantly differ between controls’ residence (data not shown). Power analysis indicates that about 4,000 cases and controls would be required to find a statistically significant difference at this prevalence of schistosomiasis.

Both schistosomiasis and Buruli ulcer are very local in nature; one village can have substantial numbers of cases whereas the next village could have none. *S. haematobium* foci with infection prevalence >50% do exist in Benin but in different settlements from the Buruli ulcer foci. Should a Buruli ulcer focus coincide with a schistosomiasis focus with a higher prevalence of infection, some association between the two diseases could appear.

Detailed clinical information was available for 36 patients tested for *S. haematobium*. In all cases, at least two of four laboratory tests were positive for *M. ulcerans*. These tests were: 1) acid-fast bacilli in a smear stained by the Ziehl-Neelsen technique, 2) positive culture of *M. ulcerans*, 3) histopathologic examination of a tissue specimen, and 4) positive polymerase chain reaction (PCR) for *M. ulcerans* DNA. Five patients had confirmed infection in bone samples, so they were classified as osteomyelitis patients. Two of these five had concurrent *S. haematobium*, compared to no cases in nonosteomyelitis patients; a Fisher exact test showed this difference to be significant (p < 0.02).

These limited and preliminary data are consistent with the relationship between leprosy (caused by a mycobacterium related to the one causing Buruli ulcer, *M. leprae*) and concurrent helminth infections. The severity of leprosy has recently been linked to intestinal helminth infection, whereas the presence or absence of leprosy has not (*7*). The cytokine environment created by helminth infection may facilitate disease progression to a more severe form, or severe mycobacterial disease and helminth infection may have a common risk factor.

We were unable to furnish evidence of a link between the presence or absence of *S. haematobium* infection and Buruli ulcer, but concurrent infections could influence Buruli ulcer clinical manifestation and disease severity.
